# Lipidomic Profile Analysis of Lung Tissues Revealed Lipointoxication in Pulmonary Veno-Occlusive Disease

**DOI:** 10.3390/biom12121878

**Published:** 2022-12-14

**Authors:** Spiro Khoury, Antoine Beauvais, Jenny Colas, Anaïs Saint-Martin Willer, Frédéric Perros, Marc Humbert, Clarisse Vandebrouck, David Montani, Thierry Ferreira, Fabrice Antigny

**Affiliations:** 1 Laboratoire Cooperatif “Lipotoxicity and Channelopathies-ConicMeds”, Universite de Poitiers, Rue Georges Bonnet, 86073 Poitiers, France; 2 Facultede Medecine, Universite Paris-Saclay, 94270 Le Kremlin-Bicêtre, France; 3 INSERM, UMR-S 999, Hôpital Marie Lannelongue, 92350 Le Plessis-Robinson, France; 4 Assistance Publique-Hôpitaux de Paris (AP-HP), Service de Pneumologie et Soins Intensifs Respiratoires, Centre de Référence de l’Hypertension Pulmonaire, Hôpital Bicêtre, 94270 Le Kremlin-Bicêtre, France; 5 PReTI Laboratory, University of Poitiers, 86073 Poitiers, France

**Keywords:** PVOD, phospholipids, lysophosphatidylcholine

## Abstract

Pulmonary veno-occlusive disease (PVOD) is a rare form of pulmonary arterial hypertension (PAH) occurring in a heritable form (hPVOD) due to biallelic inactivating mutations of *EIF2AK4* (encoding GCN2, general control nonderepressible 2) or in a sporadic form in older age (sPVOD), following exposure to chemotherapy or organic solvents. In contrast to PAH, PVOD is characterized by a particular remodeling of the pulmonary venous system and the obliteration of small pulmonary veins by fibrous intimal thickening and patchy capillary proliferation. The pathobiological knowledge of PVOD is poor, explaining the absence of medical therapy for PVOD. Lung transplantation remains the only therapy for eligible PVOD patients. As we recently demonstrated, respiratory diseases, chronic obstructive pulmonary disease, or cystic fibrosis exhibit lipointoxication signatures characterized by excessive levels of saturated phospholipids contributing to the pathological features of these diseases, including endoplasmic reticulum stress, pro-inflammatory cytokines production, and bronchoconstriction. In this study, we investigated and compared the clinical data and lung lipid signature of control (10 patients), idiopathic PAH (7 patients), heritable PAH (9 *BMPR2* mutations carriers), hPVOD (10 *EIF2AK4* mutation carriers), and sPVOD (6 non-carriers) subjects. Mass spectrometry analyses demonstrated lung lipointoxication only in hPVOD patients, characterized by an increased abundance of saturated phosphatidylcholine (PC) at the expense of the polyunsaturated species in the lungs of hPVOD patients. The present data suggest that lipointoxication could be a potential player in the etiology of PVOD.

## 1. Introduction

Cell membranes are mainly constituted of phospholipids (PLs) as combinations of several classes and molecular species, such as phosphatidylcholine (PC), phosphatidylethanolamine (PE), phosphatidylinositol (PI), or lysophosphatidylcholine (LPC), which differ as a function of their polar head group, but also according to the length and degree of unsaturation of their acyl chains [1]. PLs acyl chains vary in the number of double bonds and therefore, can be either saturated (no double bond) or unsaturated (one to six double bonds). In animals and plants, PC is the most abundant PL class, regardless of the organ or the cell type, and is also the PL that displays the widest variety in terms of acyl chain composition [2]. Variations in PL saturation levels regulate membrane biophysical properties, including fluidity, elasticity, or width. Therefore, PL saturation homeostasis is essential to sustain optimal membrane properties, which are crucial for virtually all cellular processes. Lipointoxication is a pathological state characterized by an accumulation of saturated fatty acyl chains within PLs at the expense of unsaturated species, inducing significant consequences regarding cell organelle function [3]. We recently demonstrated that respiratory diseases, such as chronic obstructive pulmonary disease (COPD) or cystic fibrosis (CF), exhibit lipointoxication signatures characterized by excessive levels of saturated PLs, leading to endoplasmic reticulum stress, interleukin-8 secretion, and bronchoconstriction [4]. Lipointoxication could be considered a new actor in the pathogenesis of respiratory disease, with high potential as a new therapeutic target.

Pulmonary hypertension (PH) is defined as an increase in blood pressure in the pulmonary artery, with a mean pulmonary arterial pressure (mPAP) >20 mmHg [5]. In the present study, we focused on Group 1 of PH. Pulmonary arterial hypertension (PAH) is a rare form of PH due to the progressive obstruction of distal PA, leading to right ventricular failure and, ultimately, death [5]. PAH can be either idiopathic (iPAH, where the cause is unknown) or heritable (hPAH) due to mutations in predisposing genes (mainly in *BMPR2*), induced by drugs or toxins, or associated with overt features of venous/capillaries (pulmonary veno-occlusive disease, PVOD). PVOD represents a rare form of PAH characterized by the preferential involvement of the pulmonary venous system and the obliteration of small pulmonary veins by fibrous intimal thickening and patchy capillary proliferation. PVOD is due to biallelic mutations in *EIF2AK4* (eukaryotic translation initiation factor 2 alpha kinase 4) encoding for general control nonderepressible 2 (GCN2). A sporadic form of PVOD (sPVOD) is described mainly in the context of exposure to specific chemotherapeutic agents or organic solvents [6,7]. In contrast to PAH, the pathobiology knowledge of PVOD is poor.

## 2. Materials and Methods

### 2.1. Patients

We retrospectively reviewed clinical data and histological samples from 10 controls, 7 iPAH, 9 hPAH (*BMPR2* mutations), 10 hPVOD (*EIF2AK4* mutations), and 6 sPVOD (no carriers mutation) patients transplanted between 2004 and January 2015. The diagnosis of either PVOD or PAH was made during interdisciplinary meetings with pulmonologists, radiologists, and pathologists. The patients used in the study were already used in our previous study analyzing the pulmonary vascular remodeling patterns and expression of GCN2 in PVOD patients [8]. The patients were part of the French Network on Pulmonary Hypertension, a program approved by our institutional ethics committee, and provided written informed consent (Protocol N8CO-08-003, ID RCB: 2008-A00485-50, approved on 18 June 2008). All human tissues were obtained with written informed consent from transplant recipients or families of organ donors, in accordance with the Declaration of Helsinki.

### 2.2. Clinical Assessment

Patient demographics, pulmonary function testing, and hemodynamics at diagnosis were obtained from the National Reference Center of Pulmonary Hypertension, Department of Pulmonology and Intensive Care Unit for Respiratory Diseases’ pulmonary hypertension database Hôpital Bicêtre, AP-HP, Kremlin-Bicêtre, Paris, France.

### 2.3. Chemical and Lipid Standards

Chloroform (CHCl_3_), Methanol (CH_3_OH), and Formic acid (HCOOH) were purchased from Sigma Aldrich (Saint Quentin Fallavier, France). Water (H_2_O) used for lipid extraction was of Milli-Q quality. Lipid standards were purchased from Avanti Polar Lipids via Sigma Aldrich (Burlington, MA, USA).

### 2.4. Lipid Extraction

The extraction of lipids from the lung samples was adapted from the Folch method [9]. Briefly, each frozen sample was first submitted to three rounds of grinding using a Precellys Evolution homogenizer (Bertin Technologies, Montigny-le-Bretonneux, France) and resuspended in 1 mL of water. The samples were then transferred into glass tubes (Pyrex Labware) containing glass beads (diameter 0.3–0.4 mm; Sigma-Aldrich, Saint-Louis, MO, USA) and vortexed for 1 min. Lipids were extracted using CHCl3/CH3OH (2:1, *v*/*v*) and shaken with an orbital shaker (IKA^®^ VX^®^ basic Vibrax^®^, Sigma-Aldrich) at 1500 rpm for two hours. The final organic phase was evaporated to dryness under a stream of nitrogen, re-dissolved in CHCl_3_/CH_3_OH (1:2, *v*/*v*), and stored at −20 °C until further analysis.

### 2.5. Lipid Analysis by Electrospray Ionization-Mass Spectrometry (ESI-MS)

Lipid extracts were analyzed by direct infusion on a SYNAPT^TM^ G2 High Definition Mass Spectrometer HDMS (Waters Corporation, Milford, MA, USA) equipped with an Electrospray Ionization Source in positive ion mode. Samples were supplemented by 1% (*v*/*v*) formic acid for positive ESI experiments. The flow rate was 5 µL/min. All full scan MS experiments were acquired in profile mode over 1 min, with a normal dynamic range from 300 to 1200 *m*/*z*. The relative abundance of each lipid species of a specific lipid class was calculated as the ratio of its MS signal to the sum of all detected MS signals in this class. MS spectra were recorded with MassLynx software^©^ (Version 4.1, Waters Corporation, Milford, USA). Data processing was carried out by the ALEX pipeline [10], with the help of Lipid Maps Lipidomics Gateway^®^ (https://www.lipidmaps.org/, accessed on 14 November 2022).

### 2.6. Statistical Analysis

Unless otherwise expressed, quantitative variables were presented as mean ± SEM. After assessment with the Shapiro–Wilk, Kolmogorov–Smirnov, D’Agostino–Pearson test, and the Anderson–Darling normality tests to determine whether the sample data followed a normal distribution, differences between the two groups were assessed using an unpaired *t*-test or a Mann–Whitney U test. Comparison between more than two groups was analyzed using ANOVA or Kruskal–Wallis tests, followed by Tukey’s or Dunn’s tests, respectively, according to the normality of the distribution. P values less than 0.05 were considered to reflect statistical significance. Statistical analysis was performed using Graphpad Prism 9 (Graphpad Software, San Diego, CA, USA).

## 3. Results

In this study, we explored the lipidomic profile of explanted lungs from 10 controls, 7 iPAH, 9 hPAH (*BMPR2* mutations), 10 hPVOD (*EIF2AK4* mutations), and 6 sPVOD (no carriers mutation) patients. Our patient cohort was predominantly male (control 6:3; iPAH 5:4; hPAH 5:4; hPVOD 5:3; sPVOD 5:3; men:women ratio, respectively). Age, body mass index (BMI), hemodynamics parameters (mPAP, cardiac index, and pulmonary vascular resistance), and 6 min walk distance (not shown) at diagnosis did not differ between all PH groups (Table 1). As expected, hPVOD and sPVOD patients displayed a lower diffusing lung capacity for carbon monoxide (DLCO/VA) than did iPAH and hPAH patients (Table 1) [7], without a change in PaO_2_ (Table 1). Apart from the control, all patients received similar PAH tri-therapy based on endothelin receptor antagonist (ERA), phosphodiesterase 5 (PDE5) inhibitor + prostacyclin intra-venous administration. It is noted that 22% of the control patients received chemotherapy before the surgical procedure, while only one had sPVOD. Finally, the gender ratio was comparable for all patient conditions (Table 1).

**Figure 1:**
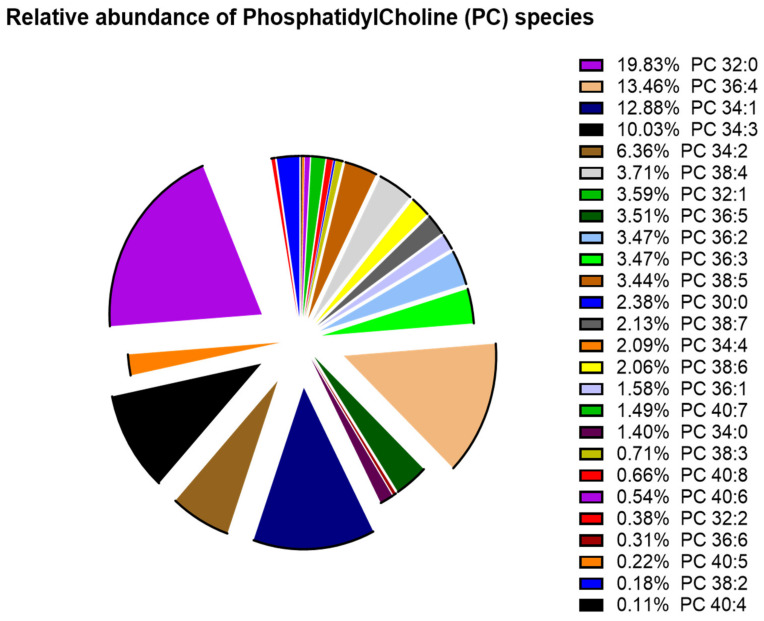
**Relative abundance of phosphatidylcholine (PC) species in lungs from control.** After total lipids were extracted, the relative abundance of phosphatidylcholine (PC) species was analyzed by mass spectrometry.

Total lipids were extracted from the explanted lungs of the various patients, and phospholipid species were analyzed by mass spectrometry. Since we previously demonstrated that patients suffering from respiratory diseases developed lipointoxication characterized by an accumulation of saturated acyl chains within PC species [4], we determined the abundance of each PC species (relative to the total lipids in the PC class) in the control patients (Figure 1). The total carbon chain length (x) and the number of carbon–carbon double bonds (y) of the various PC molecular species (x:y) are indicated.

The human lung contains remarkably high amounts of PC species, with six species representing the largest amount (around 62.5%). Among these six most represented species, PC32:0 appears as the major species, with PC32:0 (19.83%), PC36:4 (13.46%), PC34:1 (12.88%), PC34:3 (10.03%), and PC34:2 (6.36%), while other 20 PC species represent only 37.4% of PC species (Figure 1).

**Figure 2:**
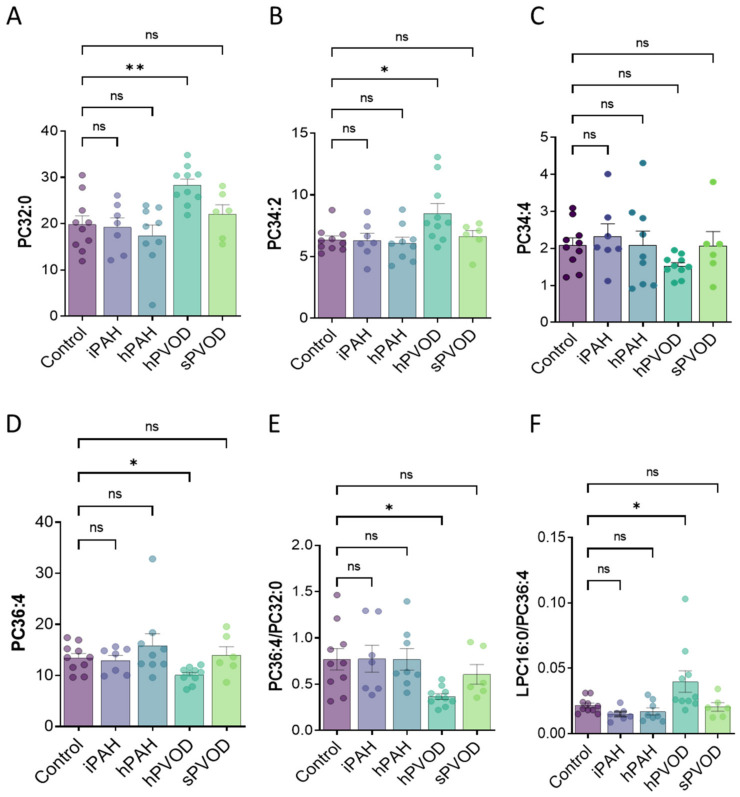
**hPVOD lung tissues are characterized by lipointoxication.** (**A**) PC32:0; (**B**) PC34:2; (**C**) PC34:4; (**D**) PC34:1. (**E**) PC36:4/PC32:0 ratio in lungs from control, iPAH, hPAH, hPVOD, and sPVOD patients. (**F**) LPC16:0/PC36:4 ratio in lungs from control, iPAH, hPAH, hPVOD, and sPVOD patients. ns: non-significant, * *p* < 0.05, ** *p* < 0.001.

Interestingly, compared to the controls, the most critical differences in lipid signatures could be observed in the hPOVD patients. Indeed, PC32:0 (bearing two saturated chains composed of 16 carbons each (16:0)) was very abundant in hPVOD patients (1.5-fold increase) as compared to the control, but it was also found in iPAH, hPAH, and sPVOD patients (Figure 2A). We observed an increase in PC 34:2 abundance (containing a 16:0 and an 18:2 chain) in patients with hPVOD compared to other groups (a 1.5-fold increase) (Figure 2B), with no significant change in the distribution of PC34:4 (Figure 2C) or PC34:3 (not shown). These selective increases mainly occurred at the expense of the PC36:4 species, bearing a 16:0 and a 20:4 chain, in hPVOD patients (Figure 2D). No significant variations were observed in the distribution of the other 20 PC species (not shown).

Consequently, the PC36:4/PC32:0 ratio was reduced in the lungs of patients with hPVOD (Figure 2E). This reduced abundance of PC36:4 in hPVOD could be explained by a higher degradation rate of these species, which leads to the production of LPC16:0. The increase in the LPC16:0/PC36:4 ratio in hPVOD (Figure 2F) suggested a higher production of LPC16:0 in the lungs of these patients.

## 4. Discussion

Maintaining the homeostasis of saturated and unsaturated fatty acids is crucial for preserving optimal membrane biophysical properties. We recently demonstrated that the lungs of COPD and CF patients exhibit lipointoxication signatures characterized by an aberrant level of saturated PLs [4]. Here, we found that the lungs of hPVOD patients displayed a very similar phenotype, characterized by an increased abundance of saturated PC at the expense of the polyunsaturated species PC36:4. This behavior could be the result of two combined effects. First, polyunsaturated fatty acid synthesis results from a sequence of desaturation steps catalyzed by strictly oxygen-dependent enzymes [11]. Consequently, we demonstrate that oxygen scarcity in vitro results in the accumulation of saturated at the expense of unsaturated PC species in bronchial epithelial cells [12]. Accordingly, chronic hypoxia is a condition encountered in patients suffering from COPD and CF due to the deterioration of lung parenchyma.

Contrary to iPAH and hPAH patients, where DLCO is unchanged, hPVOD patients are characterized by a lower DLCO, without any change in PaO_2_ (Table 1), as observed in COPD [13] and CF patients [14], suggesting that chronic lung hypoxia could be the potential origin of lipointoxication. Hypoxemia is defined as a PaO_2_ less than 60 mmHg. We found that only sPVOD patients suffered from hypoxemia (54.2 mmHg) when compared with iPAH, hPAH, and hPAH (68.5, 72.2, and 67 mmHg respectively (Table 1) [7] patients; therefore, we concluded that lipointoxication observed in hPVOD is not due to hypoxemia, but is likely due to genetic differences between the two PVOD patient subgroups. The high LPC16:0/PC36:4 ratio appears to be characteristic of hPVOD patients and could be explained by a higher degradation rate of this species via phospholipase A2 (PLA2). Indeed, this reduced abundance of PC36:4 in hPVOD led to the production of LPC16:0. Firstly, we hypothesized that low fatty acid desaturation was related to oxygen scarcity. We eliminated this hypothesis because DLCO was similarly reduced in sPVOD and hPVOD patients, and the LPC16:0/PC36:4 ratio is unchanged in sPVOD. Our second hypothesis was to link lipointoxication to the genetic status of these different groups of patients. As hPVOD patients are deficient for the *EIF2AK4* gene from birth, while this is not the case for sPVOD patients, we hypothesized that the pathogenic *EIK2AK4* mutations resulting in a decrease in the expression of GCN2 (general control nonderepressible 2, encoded by *EIF2AK4*) could be the cause of the lipointoxication observed in hPVOD patients. GCN2 is a serine-threonine kinase reported to regulate, as well as activate, the eIF2a/ATF4 signaling pathway in response to amino acid deprivation, which regulates gene expression, including genes involved in amino acid, enzyme, and transporter biosynthesis [15]. Indeed, lung GCN2 protein expression was abrogated in hPVOD patients, while GCN2 expression continues in the lungs of most sPVOD patients [16]. GCN2 deficiency could lead to an imbalance in kinases involved in integrated stress response in favor of endoplasmic reticulum stress induction [15]. ER stress was described to activate Ca^2+^-independent phospholipase A2 by generating a sphingolipid ceramide [17]. After analyzing the molecular lung signature of hPVOD and sPVOD, in addition to the loss of GCN2 protein, we also found reduced phosphorylation of the BMPRII (bone morphogenic protein receptor II) signaling effectors SMAD1/5/8 in hPVOD patients, as opposed to patients with sPVOD [16]. SMAD1/5/8 signaling regulates several genes involved in cell proliferation, apoptosis, migration, differentiation, and extracellular matrix remodeling.

Moreover, Seong et al. demonstrated that SMAD1/5/8 signaling was also related to lipid metabolism in diet-induced obese mice [18]. In addition, TGFB1 signaling, which regulates SMAD signaling, was also found to modulate intracellular lipogenesis in mice adipose tissue [19]. Lipogenesis is a metabolic pathway that synthesizes fatty acids from glucose. We hypothesized that reduced SMAD1/5/8 activation in hPVOD lacking GCN2 could contribute to lung lipointoxication.

Furthermore, GCN2 regulates fatty-acid homeostasis, since *gcn2^−/−^* mice exhibited reduced lipid mobilization in the liver. [20]. *gcn2^−/−^* mice are protected against high-fat diet-induced hepatic steatosis by inhibiting lipogenesis and reducing oxidative stress, including srebp1c (sterol response element-binding protein 1c) and FASN (fatty acid synthase) [20]. Moreover, for *gcn2^−/−^* mice exposed to high fat diet, Liu et al. demonstrated an increase in the expression of genes involved in lipolysis and a reduced expression of fatty acid gene synthesis in the liver from gcn2*^−/−^* mice [21]. These results indicate that GCN2 deficiency could be related to a dysregulation of global lipid homeostasis. We hypothesized that since birth, EIF2A4/GCN2 deficiency in hPVOD could partly cause lung lipointoxication. Since we found similar lipointoxication in hPVOD, COPD, and CF patients, it could be interesting to analyze the contribution of GCN2 in COPD and pathogeneses.

Whatever the origin of these alterations, it is likely that this lipointoxication will not be without any consequences on lung function in hPVOD patients. Indeed, we have already demonstrated that similar alterations dramatically affect crucial cellular processes, both in bronchial epithelial cells (with the induction of endoplasmic reticulum stress and massive disorganization of the overall secretory pathway, ultimately leading to cell death by apoptosis [4,22]) and muscle cells (with a global reduction in cell adaptation to mechanical constraints and therefore, reduced contraction capabilities [23]). Our present results pave the way for lipointoxication observed in the lung of a patient with hPVOD. Further additional studies are needed to understand the exact mechanism and identify the implications of lipointoxication in hPVOD.

## 5. Limitations

The number of samples used in this study is relatively low (9–10 patients), but PVOD is a subgroup of a rare disease, so it was difficult to obtain more samples from these subgroups.

Concerning the hypothetical involvement of PLA2 in lipointoxication observed in hPVOD patients, further studies are needed to quantify the level of expression and the activity of PLA2 to determine the involvement of PLA2 isoforms in hPVOD pathogenesis, since the PLA2 superfamily is described to be composed of five families defined by their physiological roles, including secretory PLA2, cytosolic PLA2, lysosomal PLA2, and two major PLA2 Ca^2+^-independent groups.

Moreover, in this analysis, our cohort is predominantly male, while PAH or hPVOD are generally characterized by female predominance, contrary to the results for sPVOD. As we compared sPVOD to other forms (iPAH, hPAH, and hPVOD), we selected similar male predominance, including in control patients, to be comparable. Finally, our analyses did not find any differences between males and females.

## 6. Conclusions

To summarize, for future studies to determine the role of GCN2 deficiency in lung lipointoxication, the present data suggest that lipointoxication could be a potential player in the etiology of hPVOD, but further additional studies are needed to understand the mechanisms explaining lipointoxication in hPVOD and to identify the significance of lipointoxication in its development.

## Figures and Tables

**Table 1:** Comparison of characteristics between patients of each group.

		Control (*n* = 10)	iPAH (*n* = 7)	hPAH *BMPR2* (*n* = 9)	hPVOD *EIF2AK4* (*n* = 10)	sPVOD (*n* = 6)
Age at PAH diagnosis			30 (±14)	33.9 (±13.6)	21.5 (±6.8) ^Θ^	49.3 (±10.9) ^Θ^
Age at surgery		64.33 (±11.0)	37.44 (±12.0) ^¥^	41.33 (±11.8)	23.38 (±7.3) ^¥,Θ^	50.00 (±11.5) ^Θ^
Gender ratio (h:f)		6:3	5:4	5:4	5:3	5:3
Exposure to organic solvents		0 (0%)	0 (0%)	0 (0%)	1 (13%)	3 (38%)
Previous chemotherapy		2 (22%)	0 (0%)	0 (0%)	0 (0%)	1 (12%)
BMI (kg/m²)		22.5 (±2.9)	22.1 (±2.5)	25.3 (±8.1)	21.5 (±4.4)	24.0 (±3.2)
6 min walk test (m)			404.8 (±71.9)	411.2 (±157.8)	430.4 (±89.8)	383.7 (±165.3)
NYHA functional class						
I-II		9	3	3	1	0
III-IV		0	6	7	7	8
Mean PAP (mmHg)			63.6 (±14.7)	50.3 (±7.8)	55.8 (±11.0)	49.6 (±18.8)
PVR (Wood units)			10.0 (±4.4)	7.5 (±2.3)	10.7 (±4.5)	9.1 (±4.6)
CI (l/min/m2)			3.6 (±1.2)	3.0 (±0.9)	2.6 (±0.8)	2.6 (±0.4)
DLCO (% pred.)		60.2 (±21.7) *n* = 5	61.2 (±18.2) *n* = 5	59.5 (±17.5) *n* = 6	52.0 (±23.2) *n* = 4	31.8 (±5.9) *n* = 4
DLCO/VA (%pred.)			67.0 (±14.2) ^£^ *n* = 4	73.3 (±15.8) ^Ʈ^ *n* = 7	31.3 (6.1) ^£^ *n* = 6	44.1 (±8.5) ^Ʈ^ *n* = 7
PaO_2_ (mmHg)			68.5 (±11.8)	71.2 (±20.8)	67.0 (±7.1)	54.2 (±9.6)
Elevated BNP/NT-proBNP			7 (78%)	6 (67%)	3 (38%)	5 (63%)
Treatments	ERA		7	6	6	5
	PDE5i		9	9	6	3
	i.v. P		9	8	5	3

PAH: pulmonary arterial hypertension. NYHA: New York Health Association. BMI: body-mass index. (h)PVOD: (heritable) pulmonary veno-occlusive disease. PVR: pulmonary vascular resistance. DLCO: diffusion of carbon monoxide. VA: alveolar volume. PaO_2_: arterial partial pressure of oxygen. CI: cardiac index. CO: cardiac output. PAP: pulmonary artery pressure. BNP: brain natriuretic peptide. NT-proBNP: N-terminal proBNP. ^¥^: significant difference compared to the control group, with *p* < 0.05. ^Θ^: significant difference between sPVOD and hPVOD, with *p* < 0.05. ^£^: significant difference between iPAH and hPVOD, with *p* < 0.05. ^Ʈ^: significant difference between hPAH and hPVOD, with *p* < 0.05.

## Data Availability

The authors declare that all supporting data are available within the article.
